# Perioperative Point-of-Care Ultrasound Use by Anesthesiologists

**DOI:** 10.7759/cureus.15217

**Published:** 2021-05-24

**Authors:** Abdullah Naji, Monica Chappidi, Abdelwahab Ahmed, Aaron Monga, Joseph Sanders

**Affiliations:** 1 Anesthesiology and Perioperative Medicine, Oregon Health Science University Hospital, Portland, USA; 2 Anesthesiology, College of Osteopathic Medicine of the Pacific, Western University of Health Sciences, Pomona, USA; 3 Internal Medicine, Northwestern Memorial Hospital, Chicago, USA; 4 Anesthesiology, Henry Ford Health System, Detroit, USA

**Keywords:** ultrasound in anesthesiology, point-of-care-ultrasound, ultrasound (u/s), anesthesia, anesthesiologists, echocardiography, bedside ultrasound, perioperative

## Abstract

Point-of-Care ultrasound (POCUS) is the bedside utilization of ultrasound, in real-time, to aid in the diagnosis and treatment of patients. Image acquisition from POCUS utilization by anesthesiologists involves the assessment of multiple organs in different perioperative situations. POCUS can be utilized to enhance clinical decision-making in a variety of perioperative situations due to its ability to assess endotracheal tube placement, cardiac function, pulmonary function, aspiration risk, hemodynamics, vascular access, and nerve visualization for regional procedures. The mounting clinical evidence for the value of POCUS in perioperative settings, its growing affordability, and its low associated risks are responsible for the nationwide movement across many anesthesiology residency programs to increase the focus on perioperative ultrasound training. The purpose of this review is to present to current anesthesiologists and anesthesiology trainees, a broad discussion regarding the diverse utility and importance of POCUS in perioperative settings.

## Introduction and background

Point-of-care ultrasound (POCUS) is the concept of bedside utilization of ultrasound to expeditiously receive imaging information that guides appropriate diagnoses, medical interventions, and acute procedures. Ideally, POCUS utilization should be goal-directed, quickly performed, and linked to improving patient outcomes [1]. Routine bedside evaluation of patients is an integral part of patient evaluation and involves many assessment tools. POCUS is a bedside assessment tool that has experienced dramatic innovation over the past few years. Over the past decade, these ultrasound devices have become smaller, with improved imaging quality, and with lower costs resulting in the expansion of their role in the perioperative assessment of patients by physicians [1]. Furthermore, an increasing amount of recent clinical research has demonstrated ultrasound to have lower costs, and have superior diagnostic sensitivity and specificity compared to other initial bedside assessment tools [1]. For example, cardiac POCUS has been demonstrated to be a superior initial diagnostic tool than electrocardiogram (EKG) for the evaluation of non-ST segment elevation chest pain [2]. Moreover, POCUS has been demonstrated to be superior to chest auscultation in identifying mainstem bronchial intubation [3]. POCUS has also been demonstrated to be superior, more accurate, and quicker than chest auscultation or chest radiograph for the detection of pneumothorax, pleural effusions, and consolidation in critical care settings [4].

With the increasing awareness and evidence to support the utility of POCUS in bedside assessment of patients, there has been a development of a strong movement to push for the increasing implementation of POCUS curriculum in anesthesiology residency training programs in the United States. Traditionally, anesthesiology residencies were required by the Accreditation Council for Graduate Medical Education (ACGME) to train their residents in transthoracic echocardiogram (TTE), transesophageal echocardiography (TEE), ultrasound-guided vascular access, and regional ultrasound nerve blocks [5]. As of 2019, the ACGME has also included lung ultrasound utilization for the detection of pneumothorax and pleural effusion as a milestone requirement for anesthesiology residency training [5]. While there has been a growing momentum for the use of POCUS in anesthesiology residencies, currently, there is not a single structured national POCUS curriculum utilized by anesthesiology residency training programs across the United States [6,7]. Therefore, the purpose of this review is to present to current anesthesiologists and anesthesiology trainees a broad discussion regarding the diverse utility of POCUS in perioperative settings.

## Review

Airway ultrasound

Endotracheal Tube Placement and Confirmation

Esophageal intubation is a serious cause of morbidity that may lead to neurological damage and mortality during anesthetic care. Generally, direct visualization of the endotracheal tube passing through the glottis is sufficient to support the successful intubation of the trachea rather than the esophagus. However, in some patients, the visualization of the endotracheal tube passing through the glottis is difficult. Airway POCUS imaging over the anterior neck can reliably be used to confirm successful endotracheal intubation and to recognize undesirable esophageal intubation [8]. Successful endotracheal intubation can be confirmed by inflating the endotracheal tube and using ultrasound to visualize corresponding tracheal dilation [8]. Moreover, successful endotracheal intubation will demonstrate air-mucosal interface with posterior shadowing on trans-tracheal view and bilateral pleural lung sliding on lung ultrasound [8]. The absence of tracheal dilation with endotracheal tube cuff inflation and the absence of pleural lung sliding indicates esophageal intubation. A meta-analysis involving 17 studies by Gottlieb et al. demonstrated that airway ultrasound confirmed successful endotracheal intubation with 98.7% sensitivity and 97.1% specificity in a mean time of 13 seconds by the anesthesiology operator [8]. The use of ultrasound to confirm endotracheal intubation is useful in clinical situations where capnography is not available or patient presentation involves low end-tidal carbon dioxide. In some cardiac arrest situations, in which the capnography end-tidal carbon dioxide is low, airway POCUS is a reliable tool to visualize and confirm successful endotracheal intubation [9].

Additional Airway Use

In complex airway situations, airway POCUS can be utilized to visualize the cricothyroid membrane (CTM) for emergent cricothyroidotomy procedures [10]. Kristensen demonstrated that cricothyroidotomy procedures involving the use of airway POCUS had a higher rate of success compared to the traditional palpation methods [11]. Airway POCUS is also helpful for anesthesiologists to use for visualizing anatomical structures when performing airway nerve blocks for awake fiberoptic intubations. Krause et al. demonstrated that awake fiberoptic intubations involving ultrasound-guided airway nerve blocks had superior airway and intubating conditions compared to blind techniques [12].

Cardiac

General

The use of POCUS for cardiac assessment is a valuable tool utilized by anesthesiologists in perioperative settings to provide real-time information to aid in identifying the etiology of certain hemodynamically unstable patient presentations. Cardiac POCUS can help the anesthesiologist obtain immediate insight on certain clinical conditions and consequently alter perioperative patient management. Such conditions include valvular abnormalities, biventricular function, pericardial tamponade, volume status, and cardiac ischemia (Table 1). Intraoperative POCUS use for cardiac assessment serves as an invaluable tool for the anesthesiologist to utilize to immediately identify the etiology of certain intraoperative abnormalities and swiftly manage it. For instance, patients who are experiencing intraoperative profound hypotension secondary to acute ischemia may demonstrate no abnormal changes in their intraoperative EKG, however, cardiac POCUS will reliably demonstrate ventricular wall hypokinesis/akinesis suggesting acute cardiac ischemia [13].

**Table 1:** Transthoracic cardiac POCUS views, probe position, and assessment. POCUS: point-of-care ultrasound; EF, ejection fraction; RV, right ventricle.

VIEW	PROBE POSITION	ASSESSMENT
Parasternal long-axis	Probe at 3^rd^- 4^th^ intercostal space with indicator directed to 10 o’clock position	Assesses EF, ventricular size, mitral valve, aortic stenosis or regurgitation, hypertrophic obstructive cardiomyopathy, and congestive heart failure
Parasternal short-axis	Probe at 3^rd^- 4^th^ intercostal space with indicator directed to 2 o’clock position	Assesses volume status, RV volume overload, coronary ischemia
Apical four chamber	Probe at 6^th^ midclavicular line intercostal space with indicator directed to 3 o’clock position	Assesses valvular function, atrial sizes, ventricular sizes, systolic function
Subcostal four chamber	Probe at subxiphoid space with indicator directed to 3 o’clock position	Assesses pericardial effusions, RV, tricuspid valve

Proficiency with cardiac POCUS can serve as a useful tool for anesthesiologists to utilize for preoperative cardiac assessment, and to help guide their decision regarding if certain patients require further cardiac assessment. For example, the utility of cardiac POCUS is invaluable in preoperative settings when attempting to make a decision on whether a patient presenting with low exercise tolerance and unknown cardiac history will require further cardiac evaluation. Hence, the information obtained from the cardiac POCUS assessment can be utilized to augment traditional assessment tools and physical exam findings to accurately decide if certain patients will require further cardiac evaluation from the cardiologist before the surgery. Gerlach et al. described how a patient with a known history of aortic stenosis presented for preoperative assessment with non-pertinent history, normal physical exam, and normal preoperative EKG was found to have a significant cardiac tamponade by cardiac POCUS [14]. Therefore, cardiac POCUS serves as an invaluable tool to utilize in-conjunction with traditional physical exam and assessment tools to increase the sensitivity and accuracy of identifying cardiac pathologies. The ease of learning cardiac POCUS was highlighted by studies that have demonstrated that first-year medical students trained in cardiac POCUS have outperformed experienced board-certified cardiologists who utilized physical exam only in accurately diagnosing cardiovascular diseases (75% versus 49% accuracy) [15].

**Figure 1:**
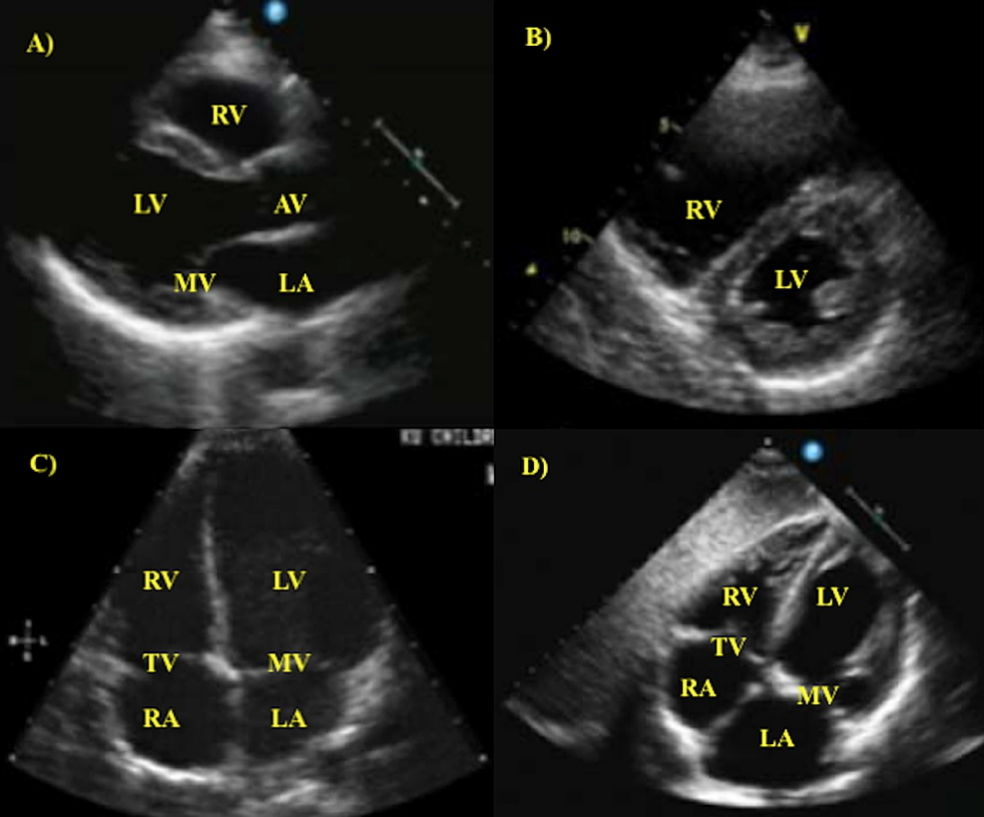
Transthoracic echocardiogram images from four cardiac views. A) parasternal long-axis view. B) parasternal short-axis view. C) apical four chamber view. D) subcostal four chamber view. RA, right atrium; TV, tricuspid valve; RV, right ventricle; LV, left ventricle; MV, mitral valve; LA, left atrium; AV, aortic valve.

POCUS use for cardiac assessment involves four views: parasternal long-axis, parasternal short-axis, apical four chamber, and subcostal four chamber (Figure 1). Volume status evaluation can be obtained from an additional subcostal view of the inferior vena cava (IVC) in the long-axis. The ideal patient positioning for obtaining cardiac views involves positioning the patient in left lateral decubitus position, which will help increase the proximity of cardiac structures to the chest wall to provide clearer ultrasound imaging. The heart is surrounded by rib structures which prevent the ultrasound waves from obtaining well-defined cardiac visualization. Hence, abducting the patient’s left arm while they are in the left lateral decubitus position increases intercostal space to provide more adequate image quality [16].

Cardiac Function

Parasternal long-axis view and parasternal short-axis view can be used to estimate left ventricular (LV) ejection fraction (EF). The subcostal four chamber view is used to estimate the size of the right ventricle (RV). Normally, the RV is smaller than the LV in the subcostal four chamber view. RV enlargement will result in a similar size of the RV to the LV in the subcostal four chamber view [17]. RV enlargement will also demonstrate flattening of the interventricular septum (“D-Sign”) when viewed from the parasternal short-axis view [17].

Cardiac evaluation with POCUS can quickly assess for ischemia. The parasternal views are frequently used to assess for cardiac ischemia, which presents as hypokinetic or akinetic wall motions. The wall motions of the LV are commonly used to assess for cardiac ischemia since the LV involves perfusion from each of the three coronary arteries at specific wall segment locations [17]. Locations of cardiac wall segments with hypokinesis/akinesis correlate to specific coronary artery vessel branch occlusion.

Cardiac POCUS can be utilized to assess valvular function and to detect valvular pathologies including regurgitation, stenosis, prolapse, and calcification [17]. The mitral valve and the aortic valve can be assessed with the parasternal long-axis view. The tricuspid valve is visualized with the subcostal four chamber view.

Pericardial effusions can most reliably be detected with the subcostal four chamber view. This view is ideal for detecting pericardial effusions since it provides a reliable visualization of the anterior pericardium, where most pericardial effusions occur [17]. Asking the patient to flex their hips and knees can reduce abdominal tension and improve the visualization quality of the pericardium.

A patient’s volume status can be assessed with both short- and long-axis parasternal views. Intravascular volume depletion is suspected with a highly-collapsible LV with a complete collapse of the end-systole LV cavity [18]. Volume status can also be assessed with IVC diameter and collapsibility visualization [17]. IVC will be visualized in the long-axis with a subcostal window. It is imperative to correctly identify the IVC and not mistake it for the aorta. The IVC can be correctly identified by visualizing the IVC emptying into the RA. The correct identification of the IVC can also be confirmed by visualizing the hepatic veins entering the IVC.

Pulmonary ultrasound

General

Pulmonary ultrasound shows many promising roles in the perioperative setting. Pulmonary POCUS has traditionally been used in critical care and emergency medicine but its use in the perioperative setting has been growing rapidly. It has even been postulated to be equal or superior to either chest radiography or chest computed tomography (CT) in some instances such as in acute respiratory distress syndrome (ARDS) [19]. Its uses include but are not limited to evaluating causes of hypoxia via pleural effusions, pulmonary edema, pneumothoraces, atelectasis, and bilateral ventilation [20,21]. It is also able to elucidate the degree of severity in pulmonary effusions and edema. Intraoperatively, the apical segments of the lung and posterior axillary line are usually accessible, which makes it a powerful tool to utilize in real-time during an operation [20]. The use of pulmonary ultrasound can be highly advantageous in the perioperative setting as compared to lung auscultation and chest radiography. One study found the diagnostic accuracy for lung auscultation, chest radiography and pulmonary ultrasound to be 61%, 47%, and 93% for pleural effusions, 36%, 75%, and 97% for alveolar consolidation, and 55%, 72%, 95% for alveolar-interstitial syndrome, respectively [19]. In addition, ultrasound examination of pleural effusion septations has been found to be superior to chest CT scan in detecting chest wall invasion by tumors (89% sensitivity and 95% specificity for ultrasound compared to 42% sensitivity and 100% specificity for chest CT scan) [22]. It is important to utilize pulmonary ultrasound as another tool in the perioperative setting.

**Figure 2:**
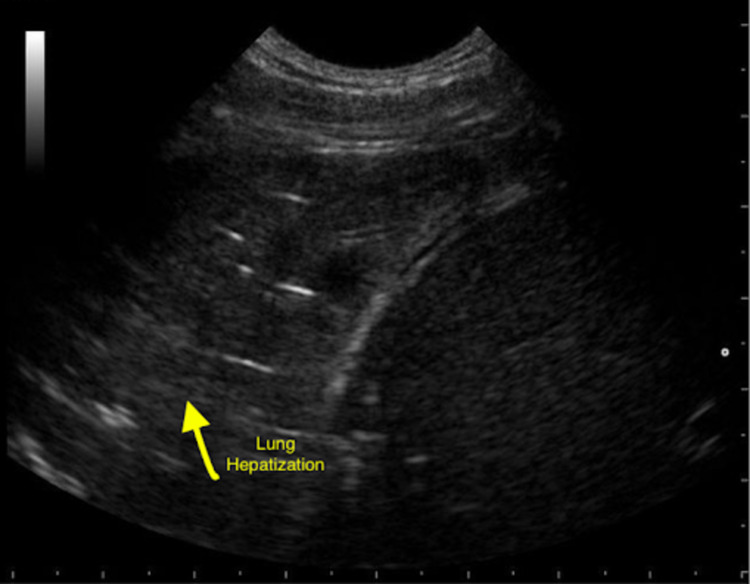
Ultrasound of the lung showing hepatization of the lung. The yellow arrow points to an area of lung hepatization representing consolidation of the lung.

There are many important findings that can be discovered using pulmonary ultrasound perioperatively. One of the most common applications intraoperatively includes the evaluation for possible pneumothorax if mechanical ventilation is not maintained, hypoxemia occurs or if asymmetrical breath sounds are auscultated. Patients are especially at risk for pneumothorax after central venous catheter placement, blunt or penetrating chest trauma or those with genetic risk factors for spontaneous pneumothorax. On pulmonary ultrasound, the presence of pneumothorax is typically indicated by the absence of lung sliding on the affected side. Other causes of the absence of lung sliding on ultrasound include pleural adhesions, breath-holding or endobronchial intubation on the opposing side. Pneumothorax can also be identified on pulmonary ultrasound by the presence of a lung point which is the transition point of which during respiration the partially collapsed lung contacts the parietal pleura. In addition, pleural effusions can also be evaluated by pulmonary ultrasound and are typically seen as an anechoic collection between the parietal and visceral pleura. Pulmonary ultrasound is useful in detecting effusions prior to performing thoracentesis. In patients with ARDS or pneumonia, pulmonary ultrasound may show the presence of lung pulse, variable lung patterns or irregularly spaced B lines. Other possible pathologies seen on pulmonary ultrasound include lung consolidation represented on ultrasound by hepatization of the lung pulmonary effusion which shows hypoechoic fluid around the lung bases, and atelectasis showing absent lung sliding but present lung pulse (Figure 2) [17].

Gastric ultrasound

Gastric ultrasound allows anesthesiologists a fast and easy way to determine a patient’s aspiration risk. Given that aspiration accounts for up to 9% of all anesthetic-related deaths, it is important to continue to improve the ways we determine aspiration risk [23]. Currently, the mainstay of decreasing aspiration risk is by using fasting guidelines prior to interventions. However, these guidelines are not without their limitations. The current guidelines by the American Society of Anesthesiologists (ASA) were intended for healthy patients undergoing elective procedures. Moreover, in some situations, the fasting status of a patient may be difficult to ascertain (i.e., emergent surgical cases, patients with altered mental status, gastroparesis, and diabetes mellitus); gastric ultrasound becomes incredibly useful in such situations [24].

By measuring the cross-sectional area of the gastric antrum using ultrasound, an anesthesiologist can accurately assess the gastric volume of their patients. This can be done while the patient is lying supine or is in the right lateral decubitus position. Gastric ultrasound is able to detect patients with a critical volume of 0.8ml/kg, helping to better determine a patient’s aspiration risk [25]. By using this technique, an anesthesiologist may feel more comfortable moving forward with anesthesia in a patient whose fasting status is unclear but with a gastric antrum cross-sectional area suggesting minimal gastric volume. More research is still needed in this area to incorporate the use of gastric ultrasound with current perioperative fasting guidelines in order to best stratify a patient’s aspiration risk.

Vascular ultrasound

Obtaining vascular access is a vital role of an anesthesiologist. Vascular access includes central venous access, peripheral venous access, and arterial access. Traditionally, landmarks were used to obtain vascular access, which can be challenging at times considering some patient’s obese body habitus, chronic medical conditions, degenerative changes, and intravenous drug use. The advancement of ultrasound technology has been able to aid anesthesiologists in obtaining various vascular access with less difficulty and fewer complications [26]. Ultrasound-guided vascular access provides numerous benefits, including visualization of the targeted vessel, reduced venipuncture attempts, reduced risk of accidentally accessing an unintended artery, visualization of anatomical variations, and avoiding thrombosed veins [27].

It is important to correctly differentiate between venous and arterial vessels, which become increasingly more difficult within deeper tissues. Venous vessels are easily compressible compared to arterial vessels when pressure is applied with the ultrasound probe. Color Doppler also serves as a useful tool to utilize in differentiating between venous and arterial vessels. Arterial vessels will demonstrate a pulsatile flow on color Doppler while the venous vessels will demonstrate a continuous flow on color Doppler [27]. Both Trendelenburg positioning and Valsalva maneuver will increase the diameter of jugular veins with minimal changes in the size of the carotid arteries, aiding the anesthesiologist in correctly identifying the internal jugular vein for central line placement.

Vascular ultrasound can also be utilized to identify deep venous thrombosis (DVT) intraoperatively or in intensive care unit (ICU) patients. The anesthesiologist will start by visualizing the common femoral vein. Moreover, all five major venous bifurcation points in the lower extremity are visualized and analyzed to rule out DVT [28]. Vascular ultrasound utilization by physicians is a reliable and accurate method to rule out DVT. A systematic review demonstrated that physicians who utilized vascular ultrasound were able to correctly diagnose DVT with 96.1% sensitivity and 96.8% specificity [29]. For vascular ultrasound examinations that are positive for DVT, the anesthesiologist can immediately proceed in utilizing cardiac POCUS to evaluate the RV for a possible formation of a pulmonary embolism [30].

Regional ultrasound

Regional ultrasound is particularly useful to the practice of anesthesia and is typically used to aid in administering peripheral nerve blocks perioperatively. It has the potential to decrease the use of general anesthesia and therefore decrease its inherent risks to the patient including a decrease in morbidity and mortality, superior postoperative analgesia, decreased postoperative complications, and improved postoperative course [31-33]. In addition, regional anesthesia such as fascial plane chest wall blocks can provide safer pain relief, provide faster functional recovery, and earlier discharge after cardiac surgery when compared to alternative methods [34].

Ultrasound guidance is invaluable as it allows for visualization of nerves, surrounding vascular or important structures, and allows for visualization of the needle tip thus preventing major complications. In comparison to nerve stimulator techniques, ultrasound guidance allows for an increased rate of successful nerve block, decreased placement time, decreased vascular puncture and local anesthetic systemic toxicity [35]. There are a wide range of peripheral nerve blocks that can be performed including upper extremity, lower extremity, scalp, cervical plexus, thoracic nerve, abdominal nerve and pudendal nerve blocks. The utility of these blocks is invaluable and ultrasound guidance is used in the majority of these blocks.

The general principles for peripheral nerve blocks remains more or less the same. The goal for peripheral nerve blocks is to identify the nerve and inject local anesthesia around the nerve. For peripheral blocks, visualization and identification of the nerves are key. A helpful method that should be employed to optimize visualization using ultrasound is spatial compound imaging which improves visualization of nerves, needle tips and tissue planes while reducing artifact [36]. A very important principle for peripheral nerve blocks is that the needle tip should be visualized throughout the procedure in order to avoid damaging nerves. Peripheral nerves typically show a hypoechoic polyfascicular “honeycomb” appearance while central nerves display a hypoechoic monofascicular or oligofascicular (one or few fascicles) appearance [37]. However, if the nerve bundle is unable to be visualized, the needle tip can be positioned in an area known to contain the nerves and a local anesthetic is injected into the area.

## Conclusions

POCUS is a valuable bedside tool that is increasingly utilized in perioperative settings due to its reliability, accuracy, speed, and ease of use. As reviewed above, the application of POCUS by anesthesiologists to different organ systems and to a variety of perioperative contexts, offers its own unique advantages to enhance clinical perioperative decision-making. POCUS can be utilized to confirm correct endotracheal tube placement and be utilized in complex airway situations. Cardiac POCUS provides invaluable information that aids in assessing challenging hemodynamically unstable situations. Pulmonary POCUS can identify numerous pulmonary conditions including pneumothorax, pulmonary edema, pleural effusion, and lung consolidation. POCUS is also utilized for the assessment of aspiration risk, vascular access, and ultrasound-guided nerve blocks. As highlighted by this review, the diverse utility of POCUS by anesthesiologists enriches the quality of healthcare patients receive in perioperative settings. Thus, the current and growing clinical evidence supporting the value of POCUS will continue to increase its utility in perioperative settings and its significance in aiding in perioperative clinical decision-making.
